# Increasing HIV Testing and Viral Suppression via Stigma Reduction in a Social Networking Mobile Health Intervention Among Black and Latinx Young Men and Transgender Women Who Have Sex With Men (HealthMpowerment): Protocol for a Randomized Controlled Trial

**DOI:** 10.2196/24043

**Published:** 2020-12-16

**Authors:** Kathryn Elizabeth Muessig, Jesse M Golinkoff, Lisa B Hightow-Weidman, Aimee E Rochelle, Marta I Mulawa, Sabina Hirshfield, A Lina Rosengren, Subhash Aryal, Nickie Buckner, M Skye Wilson, Dovie L Watson, Steven Houang, José Arturo Bauermeister

**Affiliations:** 1 Department of Health Behavior Gillings School of Global Public Health University of North Carolina at Chapel Hill Chapel Hill, NC United States; 2 Department of Family and Community Health School of Nursing University of Pennsylvania Philadelphia, PA United States; 3 Division of Infectious Diseases School of Medicine University of North Carolina at Chapel Hill Chapel Hill, NC United States; 4 Behavior and Technology Lab Institute for Global Health and Infectious Diseases University of North Carolina at Chapel Hill Chapel Hill, NC United States; 5 School of Nursing Duke University Durham, NC United States; 6 Department of Medicine SUNY Downstate Health Sciences University Brooklyn, NY United States; 7 One Cow Standing, LLC Durham, NC United States; 8 Department of Medicine Perelman School of Medicine University of Pennsylvania Philadelphia, PA United States

**Keywords:** HIV, mHealth, smartphone, men who have sex with men, racism, transgender, Hispanic Americans, mobile phone, African American

## Abstract

**Background:**

Stigma and discrimination related to sexuality, race, ethnicity, and HIV status negatively impact HIV testing, engagement in care, and consistent viral suppression (VS) among young Black and Latinx men who have sex with men and transgender women who have sex with men (YBLMT). Few interventions address the effects of intersectional stigma among youth living with HIV and those at risk for HIV within the same virtual space.

**Objective:**

Building on the success of the HealthMpowerment (HMP) mobile health (mHealth) intervention (HMP 1.0) and with the input of a youth advisory board, HMP 2.0 is an app-based intervention that promotes user-generated content and social support to reduce intersectional stigma and improve HIV-related outcomes among YBLMT. The primary objective of this study is to test whether participants randomized to HMP 2.0 report improvement in HIV prevention and care continuum outcomes compared with an information-only control arm. We will also explore whether participant engagement, as measured by paradata (data collected as users interact with an mHealth intervention, eg, time spent using the intervention), mediates stigma- and HIV care–related outcomes. Finally, we will assess whether changes in intersectional stigma and improvements in HIV care continuum outcomes vary across different types of social networks formed within the intervention study arms.

**Methods:**

We will enroll 1050 YBLMT aged 15 to 29 years affected by HIV across the United States. Using an HIV-status stratified, randomized trial design, participants will be randomly assigned to 1 of the 3 app-based conditions (information-only app-based control arm, a researcher-created network arm of HMP 2.0, or a peer-referred network arm of HMP 2.0). Behavioral assessments will occur at baseline, 3, 6, 9, and 12 months. For participants living with HIV, self-collected biomarkers (viral load) are scheduled for baseline, 6, and 12 months. For HIV-negative participants, up to 3 HIV self-testing kits will be available during the study period.

**Results:**

Research activities began in September 2018 and are ongoing. The University of Pennsylvania is the central institutional review board for this study (protocol #829805) with institutional reliance agreements with the University of North Carolina at Chapel Hill, Duke University, and SUNY Downstate Health Sciences University. Study recruitment began on July 20, 2020. A total of 205 participants have been enrolled as of November 20, 2020.

**Conclusions:**

Among a large sample of US-based YBLMT, this study will assess whether HMP 2.0, an app-based intervention designed to ameliorate stigma and its negative sequelae, can increase routine HIV testing among HIV-negative participants and consistent VS among participants living with HIV. If efficacious and brought to scale, this intervention has the potential to significantly impact the disproportionate burden of HIV among YBLMT in the United States.

**Trial Registration:**

ClinicalTrials.gov NCT03678181; https://clinicaltrials.gov/ct2/show/study/NCT03678181.

**International Registered Report Identifier (IRRID):**

DERR1-10.2196/24043

## Introduction

### Background

Young Black and Latinx men who have sex with men and transgender women who have sex with men (YBLMT) have the highest rates of new HIV diagnoses compared with non-Hispanic White peers of the same age [1], even when engaging in fewer individual-level risk behaviors [2-4]. YBLMT face multiple stigmas, racism, and discrimination related to their sexual orientation, gender, race, ethnicity, and HIV status that constrain options for building healthy relationships and supportive networks; this contributes to social isolation, inhibiting protective behaviors (eg, consistent condom use, uptake of pre-exposure prophylaxis [PrEP], and adherence to antiretroviral therapy [ART]) [5-9]. These stigmas and discriminatory practices are institutionalized and perpetuated in the systems and environments of daily life, posing barriers to engagement in care such as regular HIV testing, PrEP and ART persistence, and attending HIV care appointments [10-17]. Stigma- and discrimination-related stressors have additive or multiplicative negative physical, social, and mental health effects at the individual, interpersonal, community, and structural levels [18-23].

Although a growing number of interventions aim to address individual- or community-level HIV stigma [24,25] or consider YBLMT intersecting identities [26-30], few focus on addressing intersectional stigma as a primary mechanism for improving HIV prevention and care [31]. Furthermore, most stigma-focused interventions are delivered in person, are not tailored for the unique developmental stages of youth and young adulthood, and focus on either HIV-positive or HIV-negative populations [27,28,32-36]. The social and logistical requirements of attending in-person interventions may not fit the realities of YBLMT, particularly if they are geographically isolated [37] or have perceived discrimination or experienced microaggressions within the health care system [15,38,39]. Segmenting participants by HIV status could further polarize individuals in the community, reinforcing rather than reducing HIV-related stigma [40,41]. The biomedical advances of PrEP and Treatment as Prevention and the *undetectable equals untransmittable* (U=U) movement necessitate more inclusive, accessible intervention approaches [42].

As a strengths-based approach, social networks hold the potential power to resist stigma and improve HIV prevention and care continuum for YBLMT [43-47]. Networks also offer a unique way to understand how stigma norms and beliefs are reinforced or deconstructed. Understanding these processes within networks is important because of the critical roles of connectivity and collective identity in resilience [48-50]. A smartphone-delivered intervention that draws on the strengths of social networks could alleviate barriers to access in-person interventions, facilitate more open dialogue addressing intersectional stigma, and encourage HIV testing and care through supportive interactions with peers across the country [51-56]. Widespread access to smartphones among YBLMT in the United States makes this modality an ideal approach for increasing intervention access and reach [37,57].

A number of mobile health (mHealth) interventions are currently being developed or adapted for YBLMT [54,58-63]; however, few explicitly address the influence of intersectional stigma on HIV care outcomes or take an inclusive approach across HIV status. To address these gaps and barriers, we created an intervention strategy focused on fostering community, providing resources, and amplifying the existing strengths and assets of YBLMT [13,49,64]. The original HealthMpowerment (HMP) intervention and mHealth platform (HMP 1.0) was designed and delivered as a 3-month intervention to increase safer sex behaviors; foster community among YBLMT; and remove logistical, financial, and psychosocial barriers to intervention engagement [58]. The HMP 1.0 intervention yielded significant reductions in condomless anal intercourse (CAI) in a randomized controlled trial (RCT) conducted in the southeastern United States [58]. The social support features of HMP 1.0 were the most frequently used intervention components, and engagement with these features was associated with improved HIV care outcomes [31]. We also found associations between engaging with stigma-related content and both psychosocial and physical health outcomes [31,65-67].

### Objectives

Building on the success and lessons learned with HMP 1.0, and with the input of a youth advisory board (YAB), we rebuilt the intervention platform to reflect participant feedback and advances in technology and design. The HMP 2.0 platform maintains strong social and informational support features and enhanced intervention components. Content focuses on fostering stigma amelioration with the goals of strengthening the continued engagement of YBLMT in HIV testing for those who are HIV negative and sustained viral suppression (VS) for youth living with HIV. Our study will also address a gap in knowledge regarding how participant engagement with mHealth interventions is linked to HIV prevention and care outcomes [68-70]. We will use paradata metrics (data automatically collected as users interact with mHealth interventions such as time stamps in system logs to quantify time spent using the intervention) to gauge how intersectional stigma- and HIV-related outcomes differ based on participants’ frequency of use (exposure), amount of time spent (engagement), and types of intervention components used (usage) on the app [71].

This intervention trial is built on the scientific premise that network interventions can support behavior change [21,22,43,45,46,72,73]. Given that YBLMT widely use web-based networking sites and apps to socialize with peers [51,53,74-76], HMP 2.0 promotes social support by creating a connected virtual community [56]. We hypothesize that compared with YBLMT assigned to an information-only app condition, YBLMT assigned to an intervention arm of HMP 2.0 will report greater changes in stigma-related measures (eg, anticipated stigma, internalized stigma) and be more successful at buffering the negative sequelae of intersectional stigma and circumventing barriers to successful engagement in the HIV prevention and care continuum [46,77]. As the structural (eg, size, density) and interactional (eg, relational roles between ties; frequency and reciprocity between actors) components of a network also affect behaviors [78-85], we will compare efficacy and engagement levels between 2 types of recruitment networks: a researcher-created network and a peer-referred network. We hypothesize that participants assigned to the peer-referral network condition will have greater success in eliciting peer social support, engaging with the intervention, and achieving the desired stigma- and HIV-related outcomes than participants in the researcher-created network condition.

## Methods

### Timeline

This project began in September 2018. The initial period of study start-up activities (eg, obtaining institutional review board [IRB] approvals, hiring and training staff, rebuilding the intervention platform structure, developing the study protocol and study-specific procedures, recruiting YAB members) lasted until September 2019. October 2019 to June 2020 consisted of working with the research team, technology partner, and YAB members to update and expand intervention content; finalize and program all study tools; create HIV and viral load self-testing multimedia support materials and shipping procedures; establish robust recruitment, retention, and engagement plans; and optimize the functionality and usability of the intervention platform and administrators’ data management dashboard. Recruitment began in July 2020 and is anticipated to complete between June and September 2022. Final participant follow-ups will end by September 2023 followed by data analysis, and the study results will be disseminated in 2024.

### Study Design

**Figure 1:**
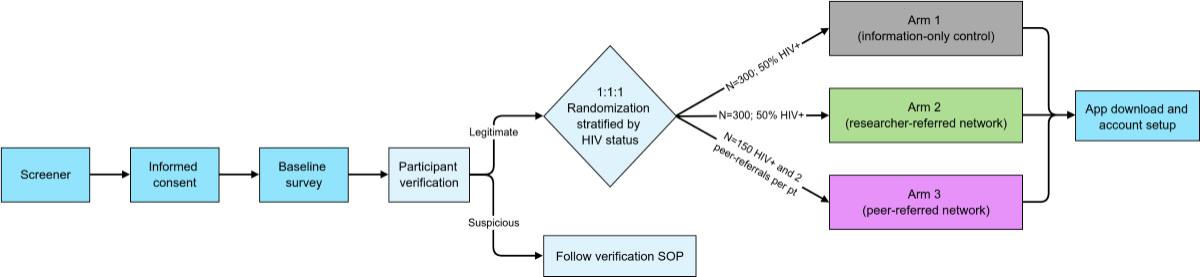
HealthMpowerment intervention enrollment and randomization procedural flow diagram. SOP: standard operating procedure.

The study design is a 3-arm, 12-month prospective RCT enrolling a total of 1050 men who have sex with men (MSM) and transgender women (TW) across the United States. The research team will recruit and enroll 750 participants; the remaining 300 will be referred by enrolled participants. Of the 750 researcher-recruited participants, 300 will be HIV negative and 450 will be HIV positive. Participants’ self-reported HIV status at baseline will be used for allocation in our HIV-status stratified randomization procedure (Figure 1). Researcher-recruited participants who self-reported as HIV negative or serostatus unknown will be randomized (1:1; n=150 per arm) into either the information-only control arm (arm 1) or the researcher-created network (arm 2). Researcher-recruited participants who self-report as HIV positive will be randomized across all 3 study arms (1:1:1; n=150 per arm). Researcher-recruited HIV-positive participants randomized to the peer-referral network arm (arm 3; n=150) can then invite peers to participate in the intervention. Up to 2 eligible referred peers may enroll per arm 3 researcher-recruited participant. These peers will be directly assigned to study arm 3 (not randomized); referred peers who enroll may not subsequently refer other peers. We estimate that approximately half of the participant-referred enrolled peers will be HIV negative or sero-unknown (Figure 1).

Study assessments will be administered at baseline, 6, and 12 months. An abbreviated version of the study assessment is administered at months 3 and 9. Consistent with best practices, we will use validated measures developed as part of the US National Institutes of Health Adolescent Medicine Trials Network for HIV/AIDS Interventions studies [86].

### HMP 2.0 Intervention

#### Theoretical Foundations

The theoretical foundation of HMP 2.0 builds on the intervention’s original behavior change theory—the Integrated Behavior Model [87]—and increases the salience of stigma-related beliefs, norms, and attitudes through new intervention content and activities, as informed by the Conceptual Framework for HIV-Related Stigma, Engagement in Care, and Health Outcomes (the *Stigma Framework*) [88]. Structural stigma and intersectionality are overarching phenomena of the Stigma Framework. Stigma, racism, and discrimination are manifested in the social and institutional structures (eg, laws, policies, norms) that create and perpetuate disadvantage for marginalized groups [89]. Intersectionality theory proposes that the social categorizations of marginalized identities (eg, gender, sexuality, race) are not distinct but rather work together to produce and reproduce inequities [90-93]. These contexts are central to HMP 2.0’s intervention approach on the premise that stigma and discrimination shape the health and health care experiences of YBLMT and should thus be a primary target of intervention.

The Stigma Framework reflects the foundational HIV stigma work [94-96] categorizing 4 types of stigma: discriminatory events and experiences (enacted stigma), perceptions of society’s stigmatizing attitudes (community stigma), expectations of future discrimination (anticipated stigma), and acceptance of negative societal attitudes as part of one’s own beliefs (internalized stigma) [88]. The HMP 2.0 intervention features tailored content and study measures across these 4 dimensions of stigma and also categorizes a fifth dimension of *challenging stigma*, that is, the acts of naming, confronting, resisting, or otherwise countering stigmatizing experiences, beliefs, narratives, practices, and perceptions [13,65]. In the HMP 1.0 trial, 75% of all participant-contributed stigma-related content could be categorized as *challenging stigma*, underscoring its importance within this virtual space [67] and its potential role in fostering resilience and resisting stigma and discrimination [50,65]. In applying the Stigma Framework, we categorize these 5 dimensions of stigma across content about race and ethnicity, gender, sexuality, and HIV.

Finally, the Stigma Framework proposes 4 types of mechanisms operating between stigma and engagement in care and HIV-related outcomes: interpersonal factors, psychological resources, mental health, and stress processes. Psychological resources include the “tools, skills, and personal identities that individuals use to cope with stressful life events” [88]. Stigma is negatively associated with these factors [97] and they may also act as buffers along the pathway to HIV-related outcomes [44]. Mental health refers to the relationship between stigma and depression and anxiety [98]. Finally, stress processes consider the biological pathways that are activated and may have a direct or mediated impact on HIV-related outcomes. A scientific premise of HMP 2.0 is that because of multiple stigmas and discrimination, stress processes are constantly activated among YBLMT. The intervention content and community-focused design aims to raise awareness of these stressors among participants, reinforce and model positive coping strategies, and ameliorate and discourage negative coping.

HMP 2.0 incorporates interpersonal-level components through its social support and social networking approach. Analyses will employ a network dynamics perspective and explore how stigma processes operate in interactions between individuals on the intervention. Psychological resources support a range of prohealth behaviors and constructs, including adherence self-efficacy, self-esteem, and coping skills. HMP 2.0 includes a substantial focus on mental health and wellness resources, activities, and discussion. A care navigator provides tailored referral support through the app.

#### YAB

Youth involvement is vital when designing and executing a youth-focused HIV intervention [5,7,10]. Alongside the research team, we convened a YAB that includes members recruited nationally through social media (Instagram, Facebook, Twitter, and Craigslist), the Adolescent Medicine Trials Network for HIV/AIDS Interventions, and community contacts with groups that serve Black or Latinx queer communities. The YAB members are diverse in terms of age (21-29 years), race and ethnicity (3 Latinx members, 6 Black members, and 1 Black Native American member), HIV status (not reported for confidentiality), sexual orientation (not reported for confidentiality), and gender identity (8 cisgender men and 2 TW). States currently represented include Georgia, Louisiana, Michigan, New York, North Carolina, and Pennsylvania.

During intervention development, YAB members met biweekly with a designated research team member (YAB coordinator), either one-on-one or through group sessions, and provided iterative feedback as intervention functionality and content were updated, study protocol decisions were considered, and study materials and tools were developed. YAB members provided written and oral feedback; study staff took detailed notes during group discussions. Members reviewed forum topics and prepopulated comments and the organization and content of the Resources section for readability, comprehension, and relevance. They also identified gaps in content and suggested edits for language, tone, and imagery. Differences in opinions and perspectives emerged; however, none were divisive. Recognizing that these differences would also likely be reflected among study participants, the research team attempted to incorporate these multiple perspectives into intervention design and content. When relevant, the team gave greater weight to options that had a stronger evidence base or were more technologically feasible. Detailed descriptive processes regarding the YAB are planned for a YAB-focused paper, including YAB co-authorship.

**Figure 2:**
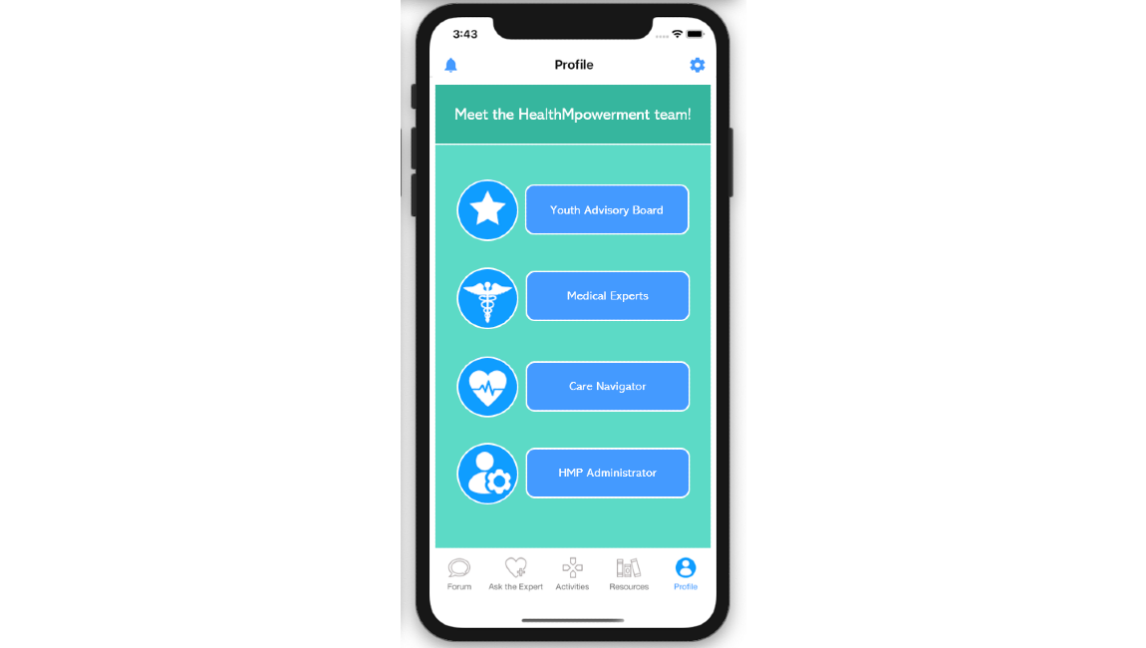
Screenshot of all icons for HealthMpowerment (HMP) 2.0 nonparticipant roles.

During the active intervention (July 2020 to June 2023), YAB members will contribute on the forums in both intervention arms (2 and 3). Their HMP usernames will be preceded with “YAB” and feature a distinct avatar icon (Figure 2) and they will help monitor the forums for ideas for new content and flag any posts that do not follow the HMP 2.0 community guidelines of mutual respect. Interested YAB members will receive additional training to support writing new content for the intervention and analyzing the participant-contributed forum conversations. YAB members are considered integral members of the study team and receive a stipend commensurate with their time contributed each month. In addition to their monthly stipend, YAB members work with a YAB coordinator to create personalized professional development plans.

Development activities range from resume and interview preparation to connecting and networking with professionals in their fields of interest. The YAB coordinator also identifies shared interests among the group and facilitates opportunities for group professional development.

#### Intervention Components

##### Intervention Features

Table 1 presents brief descriptions of the core components of HMP 2.0 aligned with the theoretical underpinnings for addressing stigma and changing behavior. Modifications to the intervention were guided by the HMP 1.0 trial evaluation [65-67], the Stigma Framework developed by Turan et al [88], the YAB study, and advances in the science of HIV prevention, care, and health communication (eg, PrEP, U=U).

**Table 1:** HealthMpowerment 2.0 core components, scientific rationale, and measurement metrics.

Features		Description	Scientific rationale (measurement metrics)
**Base app components for all study arms 1, 2, and 3**			
	Resource center	Multimedia resources and information on health and wellness, stigma, resilience, and life skills—tailored for YBLMT^a^, inclusive learning styles and health literacy Robust management system to create and tailor new content and activities	Content corresponds to and extends the HMP^b^ 1.0 intervention and aligns with the Integrated Behavior Model (number of articles read and total time spent)
	Test kit ordering	Ability to order and track HIV self-test kit and viral load self-collection test HIV test result image upload to app triggers care navigator follow-up	Provides an opportunity to get tested by reducing barriers to in-person testing (number of test kits ordered and results uploaded to app or received by laboratory)
	Care navigator	Facilitates referral and linkage to HIV services in the participant’s local community Follows up on unreported HIV test results and all shared unclear and positive results Supports app engagement and retention Troubleshoots and triages participant questions within the app to medical, study, and technology teams	Increases intervention tailoring and linkage to care for nonclinic-based study. Provides equipoise and reduces risk of social harms for remote study with HIV at-risk minors. Supports use of self-testing and self-collection for youth (number of questions asked, content of questions, and participant satisfaction)
**Interactive app components for intervention arms 2 and 3**			
	Profile	Personalized, anonymous username, avatar, brief bio, badges earned, and activities completed	Personalization, gamification, and cues to action incentivize engagement (number of times visited and profile sections completed)
	Activities	Activity templates include quizzes, self-assessments, goal setting, choose-your-own-adventure, sorting, and matching	Interactive features will enhance learning, skill building and coping skills (number of activities done and total time)
	Ask an expert	HIV, STI^c^, sex questions answered by board-certified physicians Care navigator directs participants to on- and off-app follow-up resources	Provide evidence-based answers to participants’ health questions (number of questions asked, total time, and content of questions)
	Forums	Start or add to existing discussions, upload images, videos, memes, etc Favorite posts and follow others Staff monitor and add to forum posts and include polls to encourage dialogue	Forum topics correspond to Stigma Framework constructs. Peer support for resilience and behavior change (number of posts, comments, and likes; total time; content of posts)
	Gamification	Sophisticated tracking of app use to trigger behavior-specific rewards Badges awarded for tracked events	Gamification features incentivize continued engagement (number of log-ins and badges earned)
**Peer network referral features for intervention arm 3 only**			
	Peer referral center	Unique referral code provided to share with peers Participant can track how many of their referred peers have successfully enrolled and then link to their in-app profiles	YBLMT widely use networking sites and apps to socialize with peers. Structural and relational aspects of social networks affect decision making and behaviors (number of peers referred and enrolled, characteristics, eg, size, density, and diversity of intervention networks)

^a^YBLMT: young Black or Latino men who have sex with men and transgender women.

^b^HMP: HealthMpowerment.

^c^STI: sexually transmitted infection.

##### HMP 2.0 App Intervention Study Arms

###### Information-Only Control Group (Arm 1)

**Figure 3:**
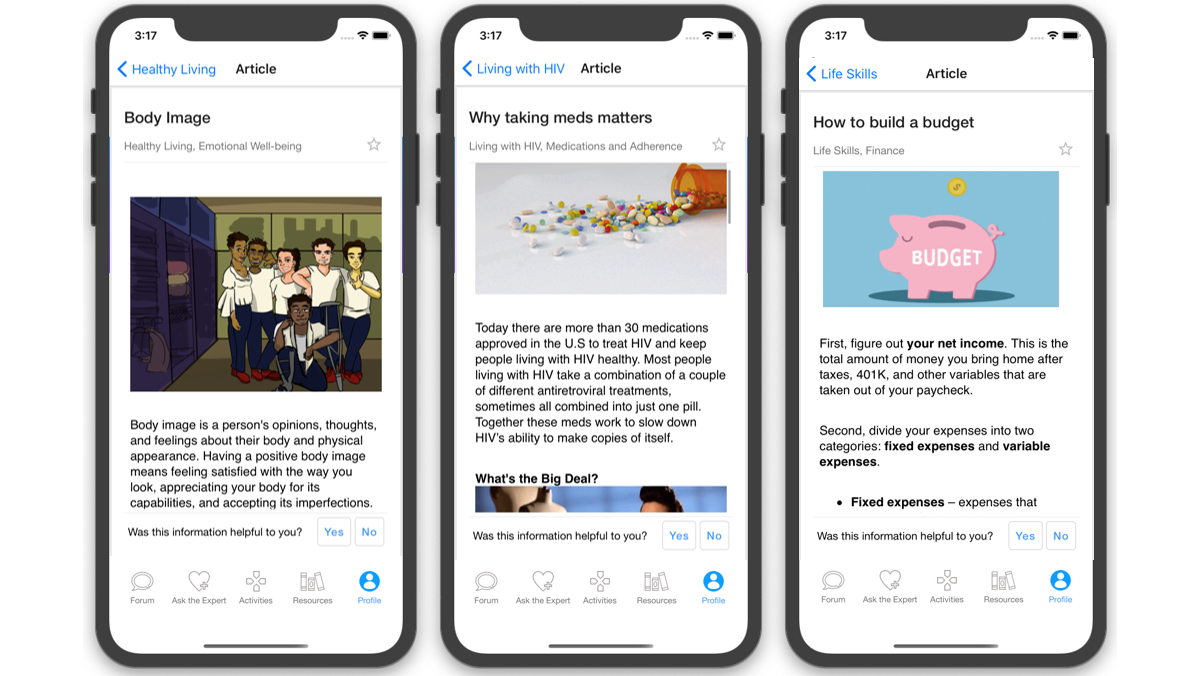
Example informational article screenshots from HealthMpowerment (HMP) 2.0 resources feature.

**Figure 4:**
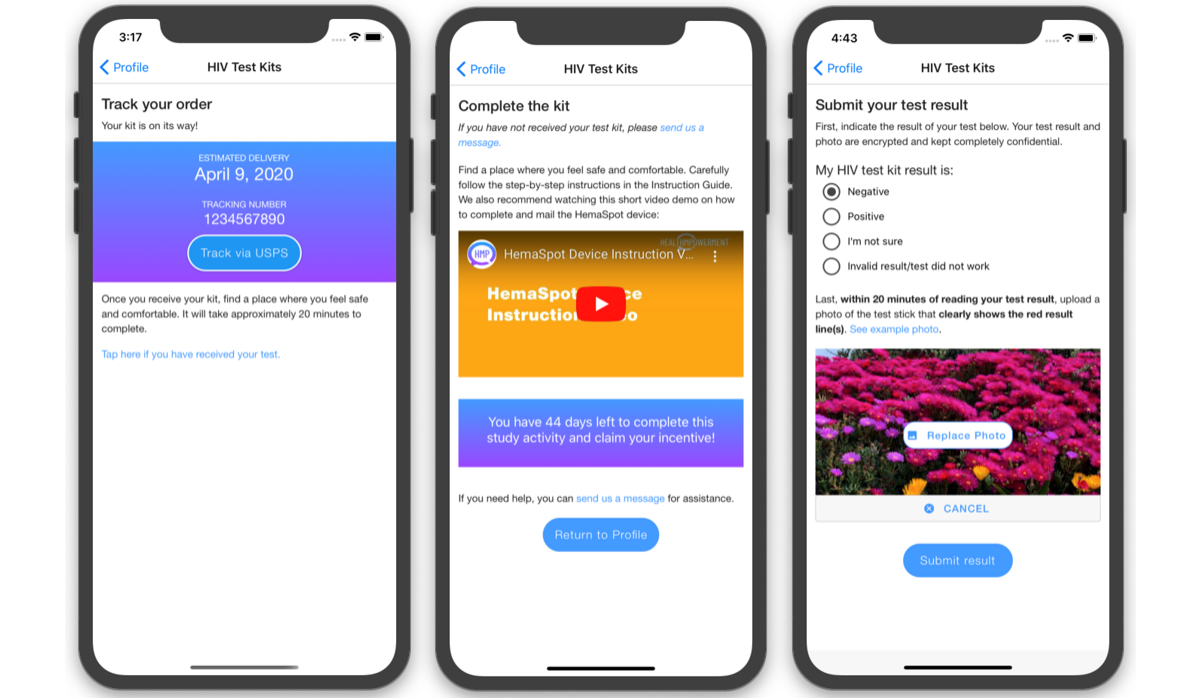
Example screenshots of the HealthMpowerment (HMP) 2.0 HIV and HemaSpot self-test kit features.

The information-only control group (arm 1) features a streamlined version of the intervention app that provides all the informational content of the Resources section (Figure 3), HIV or viral load test kit ordering and tracking (Figure 4), and access to the care navigator. Our design of the control arm balances equipoise with the research study design; in the HMP 1.0 trial, information-only control arm participants also experienced a statistically significant intervention benefit [58]. We have considered this effect in our sample size calculations.

###### Researcher-Created Network Intervention (Arm 2)

**Figure 5:**
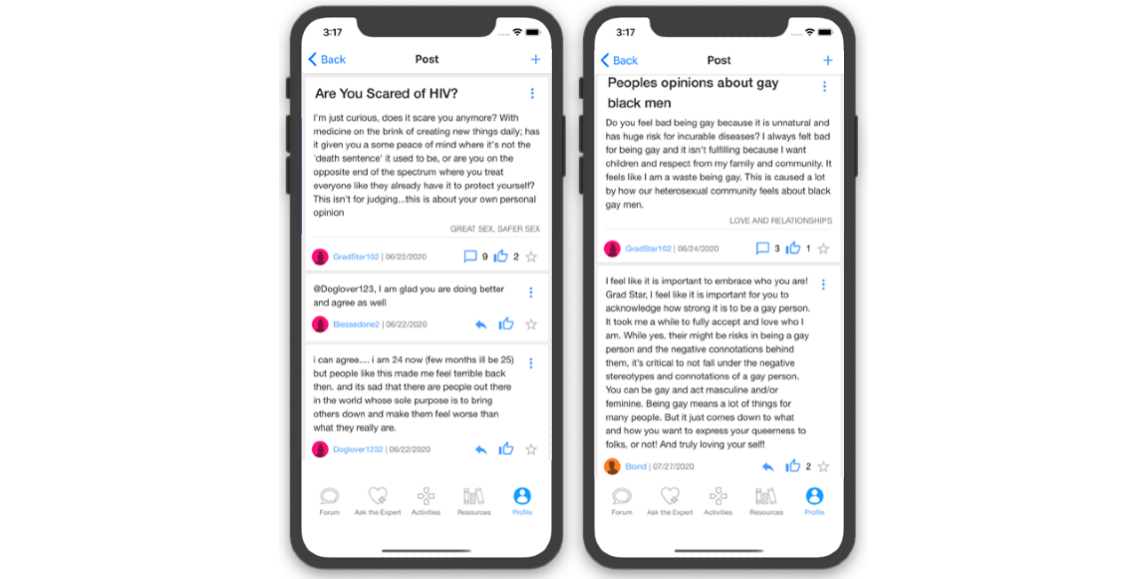
Example Forum conversation screenshots from HealthMpowerment (HMP) 2.0 intervention arms feature.

**Figure 6:**
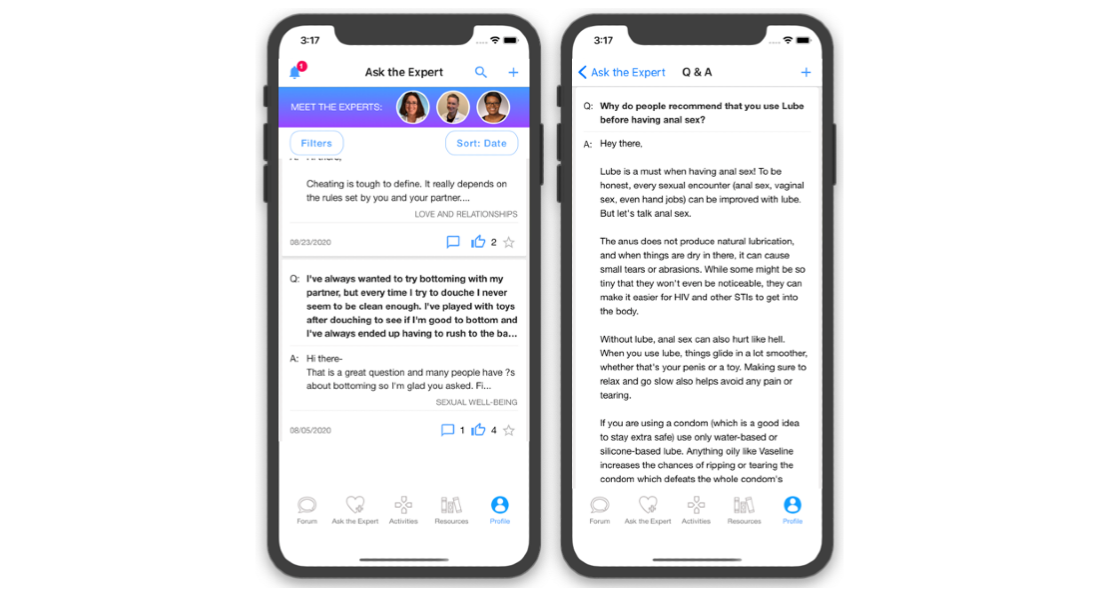
Example Ask the Expert screenshots from HealthMpowerment (HMP) 2.0 intervention arms feature.

**Figure 7:**
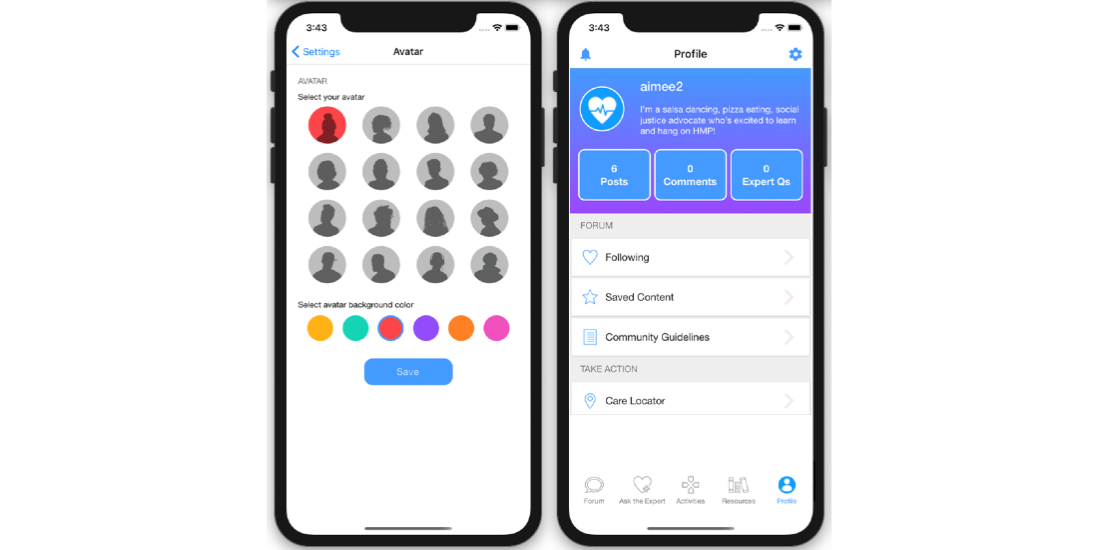
Example screenshots from avatar and personalized profile for HealthMpowerment (HMP) 2.0 intervention arms.

**Figure 8:**
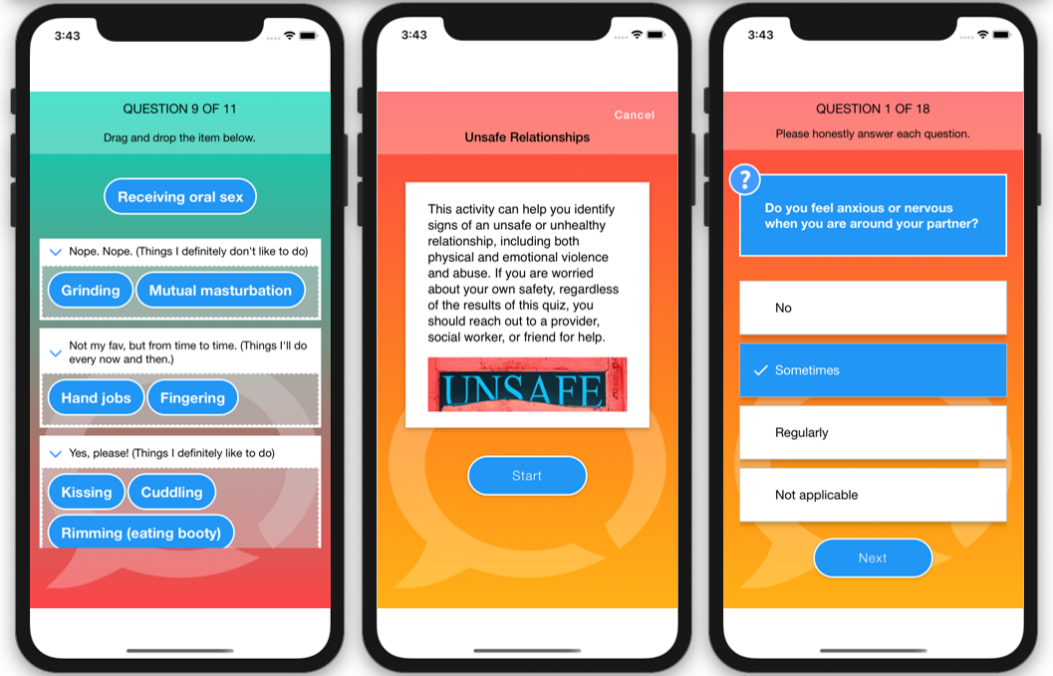
Example screenshots of decision-making activities in the HealthMpowerment (HMP) 2.0 intervention arms.

**Figure 9:**
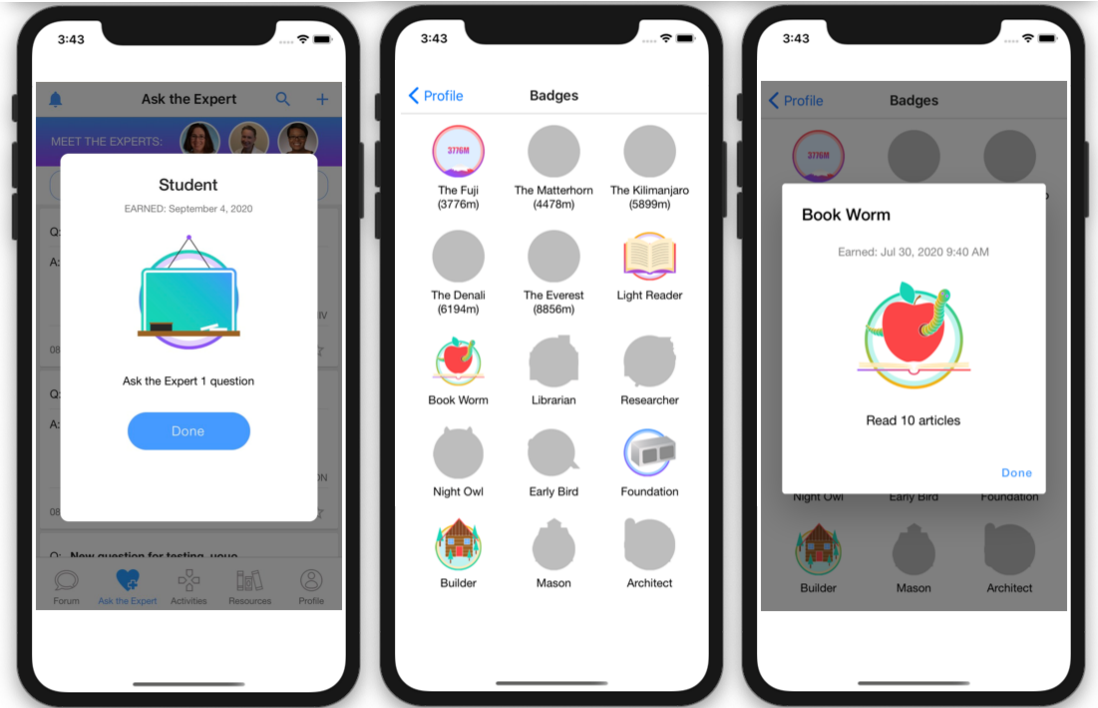
Examples of badges HealthMpowerment (HMP) 2.0 intervention arms participants can earn within the app.

Eligible, enrolled participants who are randomized to the researcher-created network intervention (arm 2) will have access to all features of HMP 2.0 (Table 1). These features include those described earlier and forums where participants can start or contribute to conversation threads and polls (Figure 5); an anonymous question and answer platform (Ask an Expert) where participants submit questions that are answered by a health care provider (Figure 6); a profile page that participants can personalize (Figure 7); an activity center supporting interactive learning, skill building, goal setting, and decision making (Figure 8); and a badge center with reward levels for all forms of engagement within the app (Figure 9).

###### Peer-Referred Network Intervention (Arm 3)

**Figure 10:**
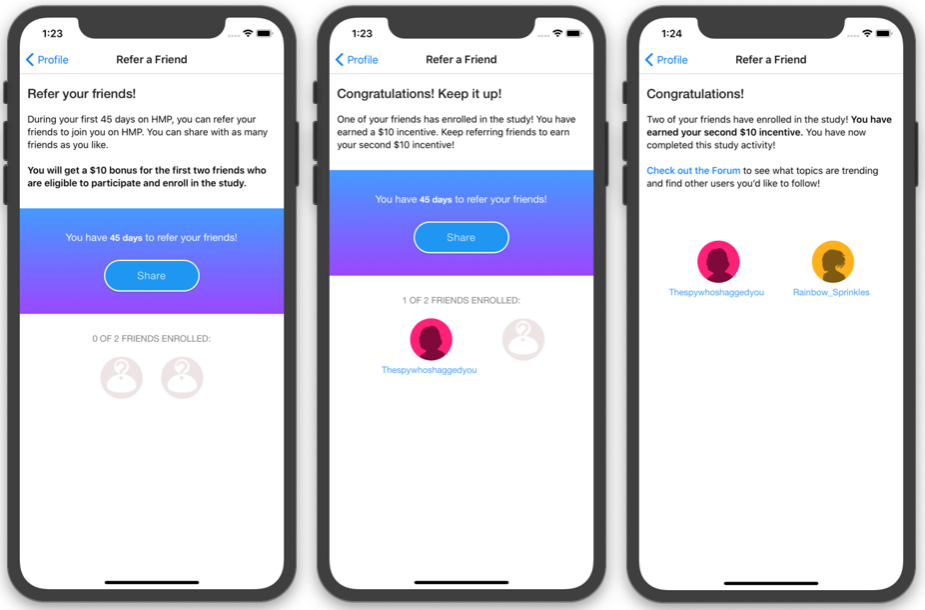
Example screenshots of peer referral feature from HealthMpowerment (HMP) 2.0 intervention arm 3.

Participants randomized to arm 3 will be enrolled into a separate, parallel version of the HMP 2.0 full-feature intervention with access to all the features described for arms 1 and 2. However, participants in arm 3 can also share a customized invitation for peers to join the study (Figure 10) [99]. Participants may continue to send invitations to peers up until their 90th study day or until 2 of their referred peers have screened eligible and enrolled in the study. At that point, the participant’s app will display a message to them that they have reached their peer referral quota and their referral button will be deactivated. In the app, participants in arm 3 can see the usernames of their referred peers but no other information about these individuals. Enrolled peers will see the username of their referrer but will not be eligible to refer peers themselves.

##### HMP 2.0 Care Navigator

HMP 2.0 provides all study arm participants access to a trained care navigator who will use existing web-based databases of resources to connect participants to trusted HIV testing and care services in their communities as needed and auxiliary resources such as behavioral health services, housing assistance programs, and food assistance programs. Within the study intervention arms (2 and 3), the care navigator will also monitor the forum and Ask an Expert health care provider platform. During all study years, the care navigator will work closely with the YAB to elicit additional content to support emerging stigmas, service-related needs, and concerns among YBLMT that can be integrated into the intervention.

##### Home Collection of Blood and Saliva for Viral Load and HIV Testing

**Figure 11:**
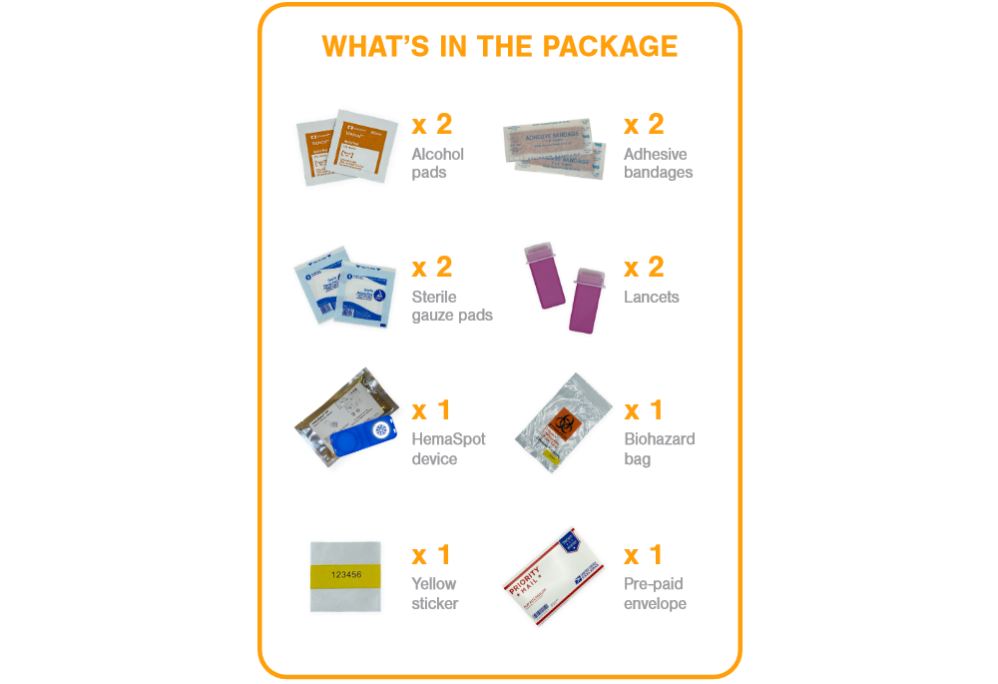
Image of participant informational package insert depicting the HemaSpot-HF blood collection kit contents.

**Figure 12:**
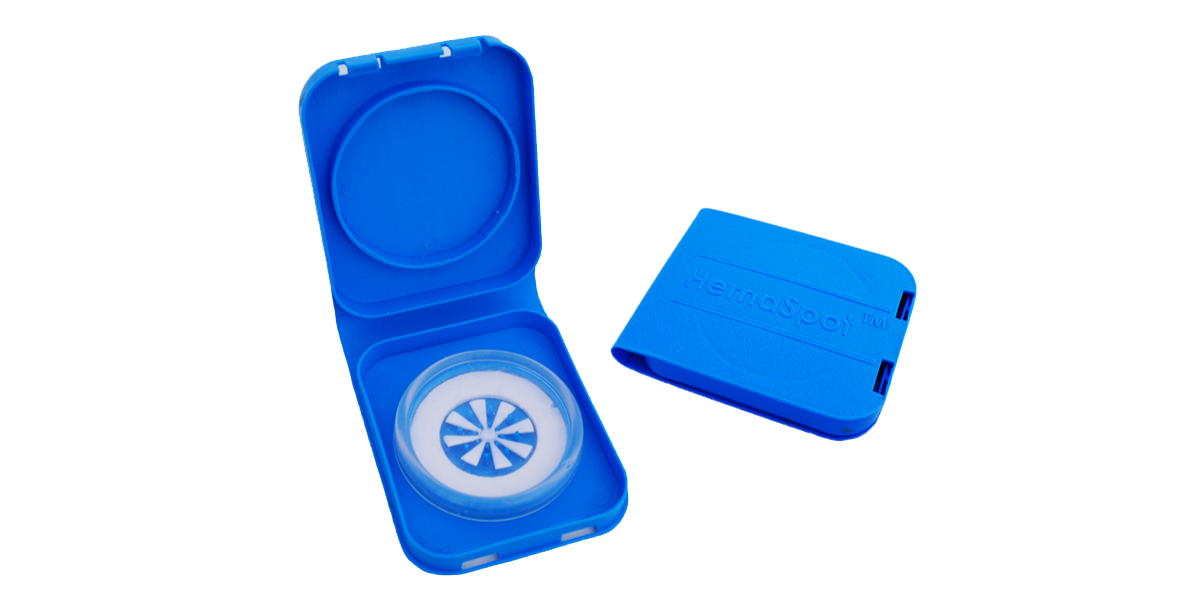
Two HemaSpot-HF blood collection devices (open and closed).

At baseline, 6, and 12 months, HIV-positive participants, across all study arms, who provide valid mailing addresses will be sent a HemaSpot-HF kit (Figure 11). The kit contains (1) one HemaSpot-HF device (Figure 12) and an instructional booklet on how to properly collect a blood specimen (eighth-grade reading level) with a link to a web-based step-by-step video; (2) a return envelope addressed to the lab with postage; (3) a lab sheet that includes the participant’s unique identification number; and (4) all materials needed to collect the blood specimen in a safe and sanitary way: alcohol pads, 2 retractable 18-gauge fingerstick safety lancets, gauze pads, adhesive bandages, and a biohazard bag.

HemaSpot-HF was developed to address technical issues associated with using traditional filter cards for dried blood spot collection [100]. Research by our team members found that men living with HIV are willing and able to collect their own blood specimens using HemaSpot-HF and may prefer this option over blood draws at clinic-based study visits as it offers convenience and privacy [101,102]. HemaSpot-HF is a small plastic device, about the size of a credit card and 0.25 inches deep. Specimens can be stored at room temperature, making HemaSpot-HF amenable to home self-collection. After blood collection (3-5 drops) with a single-use retractable safety lancet, HemaSpot-HF can be closed and shipped immediately because a desiccant in the kit quickly dries the sample inside the cartridge. Participants mail their completed kits directly to the study laboratory using a prepaid return envelope. Laboratory staff will track and store packages and test samples in batches according to their internal protocol. Participants will be informed that testing is for research purposes only and that the results of their HemaSpot-HF viral load test will not be returned to them given that we cannot guarantee clinically accurate results for individual participants because of time lags from shipment and batched specimen processing. Study staff will recommend that participants visit their regular HIV care providers or work with care navigators to access providers to obtain current HIV viral load test results.

Self-reported HIV negative or status unknown participants across all study arms can request up to 3 in-home OraQuick (R) HIV tests (oral self-swab) [103] over the course of the 12-month study. Kit requests are made via the app and are sent in a discreet, unmarked box. These rapid tests are optional and help to maintain equipoise with the viral load collection procedures with HIV-positive counterparts in the trial. We will ask participants to take a picture of their test stick that shows their test result and then upload it securely through the intervention app. In post hoc analyses, we will compare those who request home test kits with those who report traditional testing in the follow-up assessments. If a participant reports a new HIV-positive result (whether through home test kit testing results, through an HIV status change in their follow-up surveys, or by notifying the study team), the care navigator will reach out to them following a prespecified protocol to support them in making an appointment for confirmatory testing and linkage to local HIV care.

#### Participants and Enrollment Procedures

Eligible participants will be those assigned male sex at birth (no restrictions on current gender identity), aged 15 to 29 years (inclusive) at the time of screening, identify as Black, African American, Latinx or Hispanic, be a resident of the United States (verified by zip code), report at least one episode of CAI with men or TW in the previous 6 months or recruited from a partner-seeking app (eg, Grindr, Jack’d), and report having regular access to a smartphone device to access the HMP 2.0 app. Individuals who are recruited by participants assigned to the peer-referred intervention arm (arm 3) will have comparable eligibility criteria, with the exception that referred peers can identify as any race or ethnicity. Those who report testing HIV positive and having hemophilia or are currently taking anticoagulant medications will be excluded because of the risk of harm posed by the self-collected blood sampling.

The decision to include MSM and TW and Black and Latinx participants in the same study was decided early in the study design after much thought. We recognize that the experiences of stigma and discrimination manifest differently among MSM and TW communities, Black and Latinx populations, and communities living with, or affected by, HIV. Consistent with our intersectional framing, we value and acknowledge that the resultant combinations of these subgroups are even more different. In our formative study, however, we discovered that sharing these diverse experiences can bring people together to talk about these experiences and engage in inclusive dialogue that fosters support. Creating a space for open dialogue and learning facilitates individuals’ learning from each other’s differences and working through issues of stigma and discrimination they may be experiencing within the lesbian, gay, bisexual, transgender, queer community. Furthermore, some youths may still be establishing and learning about their various identities; HMP provides a safe space for broader exposure and learning, including welcoming youths who are uncertain about their sexual orientation or gender identity. Therefore, we decided that the benefits of being more inclusive in the context of this intervention outweighed the drawbacks of being more limited or targeted. All study arms have tailored content inclusive of variety in gender identity, gender expression, and sexual orientation. Similarly, the YAB and study team were attentive to include imagery and visual representations in the app that depicted diversity of gender, race and ethnicity, culture, and sexual expression. Participant avatar options feature a range of colors, hairstyles, and face shapes (Figure 7).

**Figure 13:**
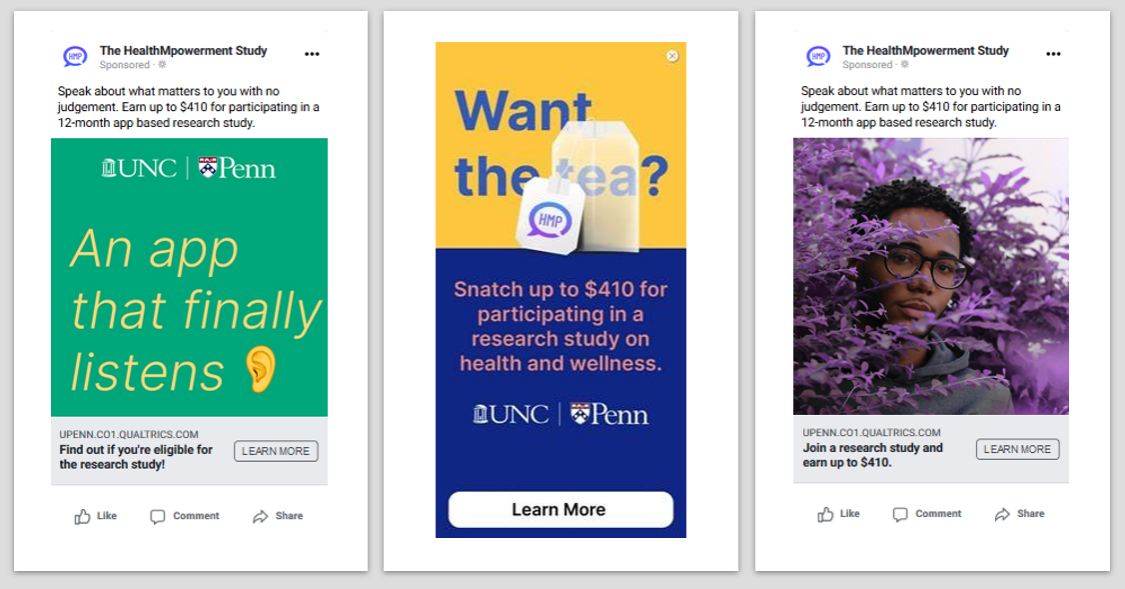
Example Facebook social media study advertisements for HealthMpowerment (HMP) 2.0 trial.

We will reach the study population using targeted advertisements based on sociodemographic characteristics on social media sites, including Facebook, Tumblr, Instagram, Black Gay Chat Live, Jack’d, Grindr, and Scruff, and through clinic referrals and participant repositories. Recruitment sites and materials have been developed alongside our YAB. Each study advertisement includes a unique link for interested individuals to complete the study screener, where they may verify their eligibility or contact the study team by email or a toll-free number (Figure 13).

We aim to enroll approximately 20 to 30 participants per month; this staggered design will allow time for the social networks to develop within the intervention. We will monitor recruitment and enrollment efforts weekly, including examining which specific study advertisements and recruitment methods (eg, social media, clinic referrals, participant repositories) generate the most enrolled participants and the characteristics of the enrolled sample. This will allow us to adapt our recruitment strategies as needed to ensure a diverse sample across age and geographic area.

The web-based, self-administered study screener explains the HMP 2.0 trial, the screening process, and confidentiality and privacy of all personal information and collects consent to complete screening. Data from those who screen eligible are reviewed biweekly for signs of fraud [104]. Individuals who pass these data checks are invited to enroll in the study. The enrollment procedures begin with an electronic informed consent form. Consented participants are then directed to a 45-min baseline survey and subsequently allocated to 1 of the 3 study arms using our HIV-stratified randomization procedure. Participants who are ineligible or do not consent are thanked and rerouted to a public site (eg, Google). A second data quality check is completed comparing select baseline and screener responses, and those who pass are sent a unique access code and directions for downloading the study app from either the Google Play or Apple app store (Figure 1).

#### Sample Size

We will enroll a total of 1050 participants from across the United States. Our sample size calculations were on the basis of pairwise HIV-stratified comparisons of the 3 groups in terms of the proportion of successful engagement using a two-sided significance level of 0.05, adjusted by the number of comparisons using a Bonferroni adjustment (significance level is 0.017 for 3 comparisons) and a minimum power of 80%. To achieve 80% power to detect a minimum effect size (odds ratio [OR] 1.9) of successful engagement in care between the 3-arm trial while maintaining the overall type I error rate at 5%, we will require at least 450 HIV-negative and 450 HIV-positive participants to detect a 17% difference. In the event of a 20% loss to follow-up, we will have sufficient power to detect an OR 2.1 (18% difference) at α=.017. Participants may continue the study even if they miss an assessment intermittently. We will compare those who completed different follow-up surveys with those who did not on key baseline predictors to check for possible sampling bias. Our analysis will use intent to treat as appropriate. The primary outcome for the proposed trial is stratified by HIV status and defined as successful engagement in care. We define power as identifying the difference in the proportions of YBLMT with sero-specific engagement in HIV care within 12 months of each of the 2 intervention arms (arm 2 researcher-created network and arm 3 peer-referred network) compared with the control arm (arm 1 information-only control), and between the 2 intervention arms 2 and 3. We have not set stratifications by gender, sexual orientation, race or ethnicity—it is set only by HIV status. We are proactively recruiting through transfocused health and community–based organizations and networks, and recruitment materials contain a variety of body images and expressions of gender and sexuality. On the basis of prior studies, we anticipate having at least 10% of our sample to identify as transgender. We will compute a post hoc power analysis to calculate the observed power to provide context for our findings for any subgroup analyses.

We will also select a purposive subsample of 45 enrolled participants (5 from the control arm; 20 from each intervention arm) across different demographic and intervention engagement profiles to complete qualitative evaluation interviews via a Health Insurance Portability and Accountability Act–compliant videoconferencing platform. A study team member will conduct interviews following a semistructured guide focused on overall intervention satisfaction, the intervention’s perceived impact on stigma and HIV outcomes, and participants’ evaluations of the social networking features (intervention arms only). Control arm participants are included in the interviews, as we expect the information-only intervention will also show a modest impact. Half of the interviews will be conducted at month 6 and half at month 12 to better understand intervention use over time and to reduce *survivor’s bias* of only interviewing participants who are retained for 12 months. Interviews will last 30 to 45 min, will be audio recorded with the participant’s consent, and will be transcribed.

#### Randomization

Participants will be allocated to the 3 study arms using a computer-generated block randomization, with stratification by HIV status. For the HIV-negative stratum (n=300, 2 treatment groups), the randomization scheme consists of 18 blocks of 8 subjects and 26 blocks of 6 subjects. Similarly, for the HIV-positive stratum (n=450, 3 treatment groups), the randomization scheme consists of 20 blocks of 9 subjects and 45 blocks of 6 subjects.

#### Engagement and Retention

In the HMP 1.0 trial, participants who remained more engaged in the intervention had greater improvements in study outcomes [58]. Thus, HMP 2.0 uses multiple strategies to promote sustained participant engagement. First, we expanded the most highly rated and frequently used features of HMP 1.0 (Forum, Ask an Expert, Resources, and Quizzes). Second, we converted the points-based reward system (which was not highly rated by participants) to a system where participants earn virtual badges for completing activities and meeting milestones within the app. We have used a similar badge system within 2 other intervention apps that young MSM have rated with high acceptability [105,106]. Third, we will follow a schedule of releasing new content onto the app each week, including activities, polls, articles, and regular YAB posts. Each month, we will also send an electronic *newsletter* tailored for intervention arms 2 and 3 that highlights new content, poll results, spotlight profiles for YAB and study staff, and the month’s most popular app discussions and articles. For overall study retention, we will follow a protocol schedule of connecting with all participants via push notifications, in-app notifications, text messages, emails, and phone calls. These will consist of preprogrammed prompts triggered after 14, 30, and 60 days of no app log-in or missed study milestone, and tailored communications from study staff.

#### Incentives

Depending on the participant’s study arm and the activities they complete, they may receive between US $390 and US $410 in e-gift cards for participating in all aspects of this study (Table 2). All participants can receive up to US $280 in e-gift cards for completing surveys at baseline, 3, 6, 9, and 12 months, with a US $50 bonus for completing at least 4 of the 5 surveys. On the basis of HIV status, participants may complete up to 3 in-home HIV tests or HIV viral load kits. Participants will receive US $20 for each (US $60 total over the 12-month study period) if they report the result of the HIV in-home test or if they complete and return the HIV viral load kits. Peer referral group participants (arm 3) can receive up to US $20 for referring eligible friends to the study: US $10 for each friend that is referred and enrolled in the study within the first 45 days. A subsample of 45 participants who are selected to complete a qualitative evaluation will receive a US $50 incentive. Incentives for completing an HIV self-test were kept intentionally low (US $20) to reduce the influence of the financial incentive driving the behavior. A critical component of the intervention is raising health awareness and individual empowerment for one’s sexual health and behaviors; thus, the incentive is a nudge to repeat the desired behavior every 3 months and is not the primary motivator.

**Table 2:** Schedule of HealthMpowerment 2.0 randomized controlled trial study incentives (US $).

Activity	Eligible participants	Baseline	3 months	6 months	9 months	12 months	≥4 surveys bonus
Complete survey	All	50	35	50	35	60	50
Return HemaSpot kit	HIV positive	20	N/A^a^	20	N/A	20	N/A
Upload HIV test result^b^	HIV negative and unknown	20	N/A	20	N/A	20	N/A
Referred peer enrolls^c^	Arm 3	20	N/A	N/A	N/A	N/A	N/A
In-depth interview	Subset of 45 across all arms	N/A	N/A	50	50	50	N/A

^a^N/A: not applicable (not all incentives are relevant for all study time points).

^b^HIV self-tests may be ordered any time during the 12-month window, up to 3 times, at least three months apart.

^c^US $10 per enrolled peer for up to 2 peers. Peer enrollment must be completed within the first 90 days following the referring participant’s enrollment.

### Outcomes

#### Primary Outcomes

Following our theoretical premise that similar stigma-related barriers impact both HIV prevention and care behaviors, we aimed to define a primary outcome of engagement in care with parallel behaviors across HIV status. For HIV-positive participants, we define successful engagement in HIV care as consistent VS across the 12-month trial (per Institute of Medicine guidelines [107]). We will ask self-reported recent (past 3 months) VS status at baseline and each follow-up assessment. Completed HemaSpot-HF kits will provide viral load biomarker data for baseline and months 6 and 12. For HIV-negative or sero-unknown participants, we define successful engagement in care as participation in routine HIV testing (2 or more HIV tests at least three months apart, per United States Centers for Disease Control and Prevention guidelines [108]). We will also examine the proportions of participants who complete at least one HIV test in the 12-month period and assess whether this is an appropriate parallel measure based on self-reported risk behaviors. Additional analyses will assess appointment and medication adherence (HIV-positive participants), uptake and maintenance of PrEP (HIV-negative participants), and testing and diagnoses for sexually transmitted infections.

#### Mediators of Intervention Effects

The secondary objective is whether participant engagement mediates the intervention effects observed in stigma and HIV care outcomes. Temporal engagement will be measured by examining participants’ number of log-ins to the app and total length of time in the app as calculated by time stamps at each log-in and logout or app timeout. We will also calculate scores for all intervention arms participants on the basis of their levels of *active* (posting and commenting) and *passive* (reading) engagement. Active engagement scores will be calculated by assigning one point for each instance a participant posted or commented on site content. Passive engagement scores will be calculated using paradata to determine the number of times they read content and then assigning one point per read. Aggregate engagement scores for each participant will be included as indicators and latent factors, as appropriate, in our analyses. This strategy will allow us to examine how different forms of engagement (eg, temporal engagement, active engagement, passive engagement) influence our study outcomes. The core measures of app engagement are included in Table 1.

An additional mediation analysis of interest is how changes in stigma and improvements across the HIV care continuum vary between the researcher-created versus peer-referred social network intervention conditions.

#### Covariates

We will measure the following constructs as potential predictors or moderators: sociodemographics (race, ethnicity, education, employment, place of birth, housing status, and history of incarceration, sexual identity, and *outness* to social network), substance use, psychological distress (depression and anxiety symptoms), intervention acceptability and use over time, use of technology and social media, and eHealth literacy [109]. These measures are included in Table 3.

**Table 3:** Study measures for HealthMpowerment 2.0 randomized controlled trial.

Variables and scale		Items
**Primary outcomes**		
	Viral suppression (HIV+)	2
	Consistent HIV testing (HIV−)	2
**Secondary outcomes**		
	Appointment adherence [110]	2
	Wilson adherence scale (HIV+) [111]	3
	PrEP^a^ uptake (HIV−) [112]	8
	STI^b^ testing and infections	3
**Experienced stigma**		
	Enacted or personalized HIV stigma scale, adapted [113,114]	12
	Gender minority stress and resilience-rejection subscale [115]	6
	Every day discrimination scale [116,117]	20
**Internalized stigma**		
	Negative self-image HIV stigma scale, adapted [114]	7
	Internalized homophobia scale—revised [118]	4
**Anticipated stigma**		
	Anticipated HIV stigma scale, adapted [114,119]	8
	Every day discrimination scale [116,117]	20
**Challenging stigma**		
	Sense of community and self-identity	20
	Stigma resistance scale, adapted [120]	16
**Contextual mechanisms**		
	Social support (select PROMIS^c^ measures) [121]	8
	Social isolation (select PROMIS measures) [121]	8
	Depression (PHQ-8^d^) [122]	8
	Anxiety (GAD^e^-7) [123]	7
	Medication adherence self-efficacy (HIV-ASES^f^) [124]	9
	Self-esteem [125]	10
**Covariates**		
	Demographics and socioeconomic characteristics	25
	Substance use (ASSIST^g^) [126]	4
	PrEP use	5
	Sexual behavior [127,128]	35
	Technology use [129,130]	24

^a^PrEP: pre-exposure prophylaxis.

^b^STI: sexually transmitted infection.

^c^PROMIS: patient-reported outcomes measurement information system.

^d^PHQ-8: eight-item patient health questionnaire.

^e^GAD: generalized anxiety disorder.

^f^ASES: adherence self-efficacy.

^g^ASSIST: alcohol, smoking, and substance involvement screening test.

### Statistical Analysis

#### Quantitative Evaluation

Clinical and demographic characteristics will be described for the entire sample and by treatment group. We will use both graphical (box-plots and histograms) and descriptive (mean, SD, IQR for continuous variables, and frequency distribution for categorical variables) approaches to describe our sample.

These will be compared with treatment groups using the analysis of variance or Kruskal-Wallis test for continuous variables and chi-square test for categorical variables. We will conduct primary analyses of our successful engagement in care outcome (month 12 status) using logistic regression models to compare each active intervention treatment group (arms 2 and 3) with the control group (arm 1) in pairwise comparison tests at an adjusted significance level of 0.017 to reduce type I errors in our 3-arm trial. This approach allows us to also test for differential efficacy between the 2 intervention conditions (ie, does the peer-referral intervention arm 3 outperform the researcher-created intervention arm 2?).

We will use the general framework of generalized linear mixed models [83,84,131] to model these longitudinal outcome trajectories [132-134]. Given that some of our outcomes are binary, some count and some continuous traits will be treated differently (identity for continuous outcome, logit for binary outcome, and natural log for count outcomes). Logistic regression analyses will be stratified by serostatus. The regression will be run with group assignment only in the model. Among HIV-positive participants, we will examine how intervention conditions influence YBLMT’s likelihood of consistent VS over 12 months. We will also test intervention effects on retention in care per Institute of Medicine guidelines (ie, proportion of HIV-positive participants who obtain at least two viral load tests [at least three months apart] within 12 months). For HIV-negative participants, we will compare the proportion of participants who obtain at least two HIV tests (at least three months apart) within 12 months across arms. Estimates will be calculated and presented with corresponding 95% exact binomial CIs.

Building on our theoretical framework, we will use structural equation modeling to test whether engagement with researcher- and participant-generated stigma content predicts changes in stigma-related outcomes over time (eg, decreases in anticipated HIV stigma) and changes in HIV care outcomes over time (eg, repeat HIV testing; consistent VS). In these HIV-stratified analyses, we will use latent class analysis to characterize participants’ engagement in the site. We will estimate the independent contributions of passive and active engagement on both stigma and HIV outcomes and how cumulative engagement in stigma-related discussions influences these outcomes. Key sociodemographic predictors will be included as covariates (eg, age, time since diagnosis). We will also conduct exploratory stratified analyses by race, ethnicity, and gender.

#### Qualitative Evaluation

Expanding on the procedures developed and successfully employed in HMP 1.0 [92], we will conduct a mixed methods analysis of participant-contributed content to the forums. All content and associated paradata of each post will be captured by the intervention database. We will use qualitative data analysis software to catalog and code all instances of stigma-related content and characterize their potential contributions to HIV risk and care behaviors. First, 2 coders will independently identify all posts that contain stigma-relevant content. Next, they will conduct a content analysis to categorize the nature of the coded stigma content as enacted, community, anticipated, internalized, or challenged stigma. Coding discrepancies will be reviewed and resolved by a third analyst. Ongoing analysis progress will be discussed during biweekly YAB meetings and feedback sought on YAB members’ interpretations of participant forum conversations and emergent themes. The final coded data set will be used for qualitative analyses and publications, and to create variables that quantify participants’ engagement with each type of stigma-related domain. These data will be used to examine how frequency of engagement with stigma content is associated with changes across HIV outcomes directly and through proposed mediators (eg, social support and isolation, substance use, depression). We successfully used this methodology and established analytic protocols to carry out these procedures [66,67,92].

For the qualitative analyses of the 45 interviews, we will create a codebook of *a priori* and emergent themes, including operational definitions of all codes and sample quotations, to illustrate how to apply each code. Two study team members will use the codebook to independently code the data, whereas a third team member will review these coded sections and resolve discrepancies. We will use qualitative data analysis software and matrices to assist with theme identification, coding textual data, and describing relationships among codes (via code co-occurrence and memoing functions) [135]. Analysis results will be used in conjunction with participants’ survey responses, biological outcomes, and usage profiles to present a mixed methods intervention results analysis.

## Results

### IRB Approval and Trial Registration

The research and ethics presented in this study were approved by the IRB of the University of Pennsylvania (829805) as the IRB of Record. Reliance agreements between the University of Pennsylvania, the University of North Carolina Chapel Hill, Duke University, and SUNY Downstate have been completed. A certificate of confidentiality was obtained from the National Institute of Child Health and Human Development, and a waiver of parental consent or assent was obtained for participants aged 15 to 17 years. This study is registered on ClinicalTrials.gov (NCT03678181).

### Recruitment and Enrollment

Study recruitment began on July 20, 2020. As of November 20, 2020, 3780 participants had completed screeners, yielding 830 eligible participants. Among the 218 participants who went on to complete the baseline survey, 94.0% (205/218) have enrolled and been randomized to a study arm, representing 19.52% (205/1050) of the total target enrollment. Enrolled participants are geographically diverse, representing all 50 contiguous United States and Washington DC, vary in age (36/205, 17.6% aged 15-19 years; 55/205, 26.8% aged 20-24 years; and 114/205, 55.6% aged 25-29 years), and 18.5% (38/205) report an HIV-positive status at baseline. Recruitment is anticipated to last through September 2022, with final study follow-up to be completed by September 2023 and results to be available in 2024.

## Discussion

### Trial Innovations

There is a great need and potential to develop, implement, and scale up HIV prevention and care interventions for YBLMT. Implementation barriers and facilitators and analyses from this study will inform the design of mHealth engagement strategies for connecting with YBLMT across the HIV continuum. Our results will further the field’s understanding of how engagement with an mHealth intervention that builds on the networks of YBLMT impacts and curtails the role of intersectional stigma in well-being. We are also testing several remote health service intervention components among young people (mail-based self-collected viral load via finger prick, HIV self-testing via oral swab, remote care navigation to confirmatory testing, PrEP, and HIV care) that have previously been tested among an older age range of predominantly White MSM.

### Principal Contributions to the Field

These results will be critically important to the field with the exponential expansion of telehealth and at-home, self-administered biomarker specimen collection and testing. Our projects’ tools and framework may inform future mHealth and stigma reduction initiatives for YBLMT.

## Abbreviations

ART: antiretroviral therapy
CAI: condomless anal intercourse
HMP: HealthMpowerment
IRB: institutional review board
mHealth: mobile health
mPI: multiple principal investigator
MSM: men who have sex with men
NIMH: National Institute of Mental Health
OR: odds ratio
PrEP: pre-exposure prophylaxis
RCT: randomized controlled trial
TW: transgender women
U=U: undetectable equals untransmittable
VS: viral suppression
YAB: youth advisory board
YBLMT: young Black and Latinx men who have sex with men and transgender women who have sex with men
